# Copy number variations and gene polymorphisms of Complement components in ocular Behcet’s disease and Vogt-Koyanagi-Harada syndrome

**DOI:** 10.1038/srep12989

**Published:** 2015-08-13

**Authors:** Dengfeng Xu, Shengping Hou, Jun Zhang, Yanni Jiang, Aize Kijlstra, Peizeng Yang

**Affiliations:** 1 The First Affiliated Hospital of Chongqing Medical University, Chongqing Key Laboratory of Ophthalmology and Chongqing Eye Institute, Chongqing, P. R. China; 2 University Eye Clinic Maastricht, Maastricht, The Netherlands

## Abstract

Complement is involved in many immune-mediated diseases. However, the association of its copy number variations (CNVs) and polymorphisms with Behcet’s disease (BD) and Vogt-Koyanagi-Harada syndrome (VKH) is unknown. We examined copy number and mRNA expression by real-time PCR. Cytokine production by stimulated peripheral blood mononuclear cells (PBMCs) in genotyped individuals was measured by ELISA. The frequencies of having more than two copies of C3 were significantly increased in BD and VKH, whereas CNV of C5 was only associated with BD. Increased frequencies of the GG genotype of C3 rs408290 and C5 rs2269067 were found in BD. No association was observed between C3 and C5 SNPs and VKH. mRNA expression in the high CNV group and GG cases of C3 and C5 was significantly higher compared to other genotypes. Increased interleukin-17 and IFN-γ was observed in the high CNV group and GG genotype cases of C3. Interleukin-17 but not IFN-γ was increased in the high CNV group and GG genotype cases of C5. No effect of C3 or C5 genetic variants was seen on the production of TNF-α, IL-10, IL-1β, MCP-1, IL-6 and IL-8. Our study thus provides further evidence for a role of complement in the pathogenesis of uveitis.

Uveitis is one of the primary eye diseases leading to blindness all over the world^1^. Behcet’s disease (BD) and Vogt-Koyanagi-Harada (VKH) syndrome are two important well defined uveitis entities that are common in Asia^2^,^3^. Behcet’s disease is an autoinflammatory disease involving multiple organ systems and manifesting with recurrent oral ulcers, genital ulcers, nongranulomatous uveitis, and skin lesions^4^, and is often associated with disorders such as arthritis, intestinal ulcers, and central nervous system lesions^5^. VKH syndrome is recognized as an autoimmune disease accompanied by a bilateral granulomatous uveitis, but also involving other organs containing melanocyte target autoantigens^6^. Although the detailed etiology and mechanism of BD and VKH syndrome remain unclear, genetic factors in the initiation and development of both diseases have been recognized since a few decades^7^,^8^.

Single nucleotide polymorphisms (SNP) and gene copy number variations are two types of human genome variation. A SNP is the most common genetic variation and is currently considered as an important marker to recognize the genetic loci that contribute to human diseases. CNV is a new kind of genetic variation describing the fact that DNA fragments can vary from 1 KiloBase to MegaBases in size. These fragments contains gene duplication, deletion, and rearrangements ranging from one hundred base pairs to several mega base pairs in size^9^. Previous studies have shown that CNV may be closely related to phenotypic variation and play a key role in the evolution and development of species^10^.

The complement system is regarded as a crucial element in innate immunity^11^. The activation of the complement system occurs along three routes named alternative pathway, classical pathway and lectin pathway. The sequential activation of the three pathways collectively leads to complement C3 activation and C5 cleavage and finally to the membrane attack complex (MAC) to be generated on the cell surface^12^. Several inflammation-mediated ocular diseases have been identified to be related to complement activation and regulation, such as age-related macular degeneration, keratitis, and uveitis^12^,^13^,^14^. In addition, many studies have confirmed that genetic variation of individual complement components may play a role in the predisposition to these ocular diseases^15^,^16^. Recent studies showed that a high C4 copy number is associated with BD and VKH syndrome^17^,^18^. Additionally, some studies have suggested that complement components C3 and C5 are involved in the pathogenesis of experimental autoimmune uveoretinitis^19^,^20^.

Whether the CNVs and SNPs of complement C3, C5 and the other downstream complement components that participate in the final pathway of the complement cascade are associated with BD and VKH syndrome has not yet been reported and was therefore the subject of the study reported here. To address this question we analyzed the copy number variations and polymorphisms of C3, C5, C6, C7, C8A, C8B and C9 in two common types of uveitis in China.

## Results

### Clinical characteristics of patients with BD or VKH syndrome

The data of clinical features, age and gender distribution of BD patients, VKH patients and controls were taken at the time of diagnosis and details are shown in Supplementary Tables 2–3.

### The association of CNVs with BD or VKH syndrome

The C3 and C5 copy number variation in patients with BD, patients with VKH and controls was calculated in three stages. When the statistical difference was significant at the first stage between patients and normal controls (P < 0.05), we repeated the analysis in a second (replication) cohort to confirm the results. At the first stage, the frequency of exceeding 2 copies of C3 was increased in BD (Pc = 5.5 × 10^−3^, OR = 3.4) and VKH (Pc = 0.018, OR = 3.1) as compared with controls. In the second cohort, a significantly increased frequency of exceeding 2 copies was found in BD and VKH versus controls (Pc = 4.2 × 10^−6^, OR = 2.9; Pc = 1.4 × 10^−5^, OR = 2.8, respectively). In the third analysis stage, we combined the data of the two cohorts and confirmed that the frequency of >2 copies of C3 was significantly increased in both BD and VKH (Pc = 5.3 × 10^−9^, OR = 3.0; Pc = 6.4 × 10^−8^, OR = 2.8, respectively). The frequency of >2 copies of C5 was increased in BD compared with controls in both the first and second cohort. Taken together, the combined data identified that more than 2 copies of C5 was a risk factor for BD (Pc = 1.1 × 10^−8^, OR = 3.4) (Table 1). The copy number variation of C5 showed no statistical difference between VKH patients and controls at the first stage (Table 1). The copy number variations of C6, C7, C8A, C8B and C9 displayed no statistical difference in BD patients and VKH patients compared with controls (Supplementary Table 4).

### Increased frequency of GG genotypes of C3 and C5 in BD patients

The frequencies of genotypes and alleles of 10 SNPs were analyzed in 980 BD patients, 378 VKH syndrome patients and 1369 healthy controls. The genotype frequencies of the ten SNPs did not deviate from HWE in normal controls (P > 0.05). The analysis was again carried out in three stages. Taking the data of two cohorts together, the frequency of the C3 rs408290 GG genotype in BD patients was significantly increased compared with controls (Pc = 1.5 × 10^−7^, OR = 2.31) (Table 2). There were no statistical differences for the other five SNPs of C3 between BD patients and controls at the first stage (Supplementary Table 5). The frequency of rs2269067 GG genotype and G allele of C5 in BD was significant higher than that in controls after combing the data from the two cohorts (Pc = 1.3 × 10^−8^, OR = 1.73; Pc = 1.4 × 10^−7^, OR = 1.52, respectively) (Table 2). The genotype and allele frequencies of the other three SNPs of C5 showed no statistical difference concerning BD patients and VKH patients as compared with controls after Bonferroni correction (Supplementary Table 6). The genotype and allele frequencies of the ten SNPs showed no statistical differences between patients with VKH syndrome and normal controls. In other words, the polymorphisms of C3 and C5 showed no association with VKH syndrome in a Chinese Han population in this study (Table 2 and Supplementary Tables 5–6).

### Stratified analysis for C3 and C5 Copy number variation and polymorphisms with main clinical features of BD or VKH syndrome

A stratified analysis was conducted to test whether copy number variation of C3 and C5, rs408290/C3 and rs2269067/C5 was associated with the main clinical features of BD or VKH syndrome. The main clinical features of BD comprised genital ulcer, arthritis, skin lesions and positive pathergy reaction. The main clinical manifestations of VKH included headache, tinnitus, vitiligo, alopecia and poliosis. No association was found for copy number variation of C3 and C5, rs408290 and rs2269067 with any clinical manifestation of BD or VKH (Supplementary Tables 7–11).

### The influence of copy number variation on C3 and C5 expression

**Figure 1:**
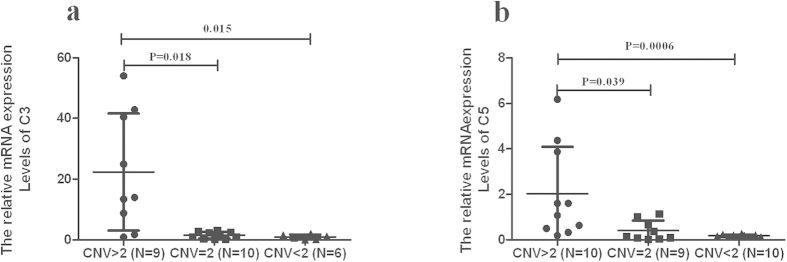
The relationship between the expression of C3 and C5 CNV. mRNA expression of C3 in PBMCs from normal controls with different copy numbers (**a**) (CNV > 2: n = 9, CNV = 2: n = 10, CNV < 2: n = 6). mRNA expression of C5 in PBMCs from normal controls with different copy numbers (**b**) (CNV > 2: n = 10, CNV = 2: n = 9, CNV < 2: n = 10). Two independent samples Nonparametric test was used to examine the significance.

As shown above, the copy number variations of C3 showed a strong association with BD and VKH syndrome, and the CNVs of C5 was shown to be associated with BD. To evaluate the mRNA expression of C3 and C5 in relation to the gene copy number, we tested PBMCs from healthy genotyped individuals. The results showed that mRNA expression of C3 in individuals having >2 copies was higher than in persons having less than 2 copies or 2 copies (P = 0.015, P = 0.018, respectively). The mRNA expression in individuals having more than2 copies of C5 was significantly increased as compared to those with 2 or fewer copies (P = 0.039, P = 0.0006, respectively) (Fig. 1).

### The influence of rs408290 on C3 expression and rs2269067 on C5 expression

**Figure 2:**
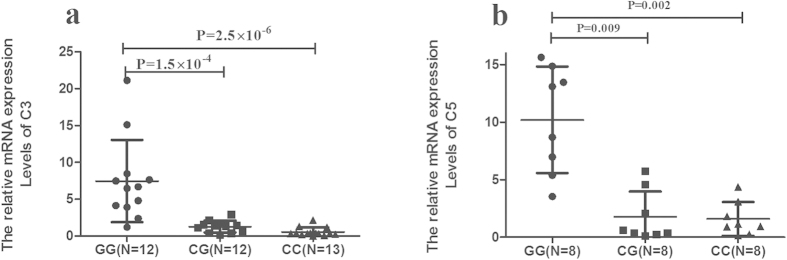
The gene expression of C3 and C5 with different genotypes. mRNA expression of C3 rs408290 and C5 rs2269067 with three different genotypes in PBMCs from healthy controls. The number of different genotype cases of C3 rs408290 (**a**) (GG: n = 12, CG: n = 12, CC: n = 13) and C5 rs2269067 (**b**) (n = 8 per group) is shown in the bracket under horizontal axis. Significance was tested by SPSS two independent samples Nonparametric test.

The aforementioned results revealed an obvious association of C3 rs408290 and C5 rs2269067 with BD. We subsequently investigated mRNA expression of C3 and C5 in PBMCs from healthy individuals with a known SNP genotype. The mRNA expression of C3 rs408290 in GG cases was significantly increased as compared to CC and CG cases (P = 2.5 × 10^−6^, P = 1.5 × 10^−4^, respectively). The mRNA expression of C5 rs2269067 in GG cases was significantly increased as compared to CC and CG cases (P = 0.002, P = 0.009, respectively) (Fig. 2).

### The influence of copy number variation of C3 and C5 on cytokine production

**Figure 3:**
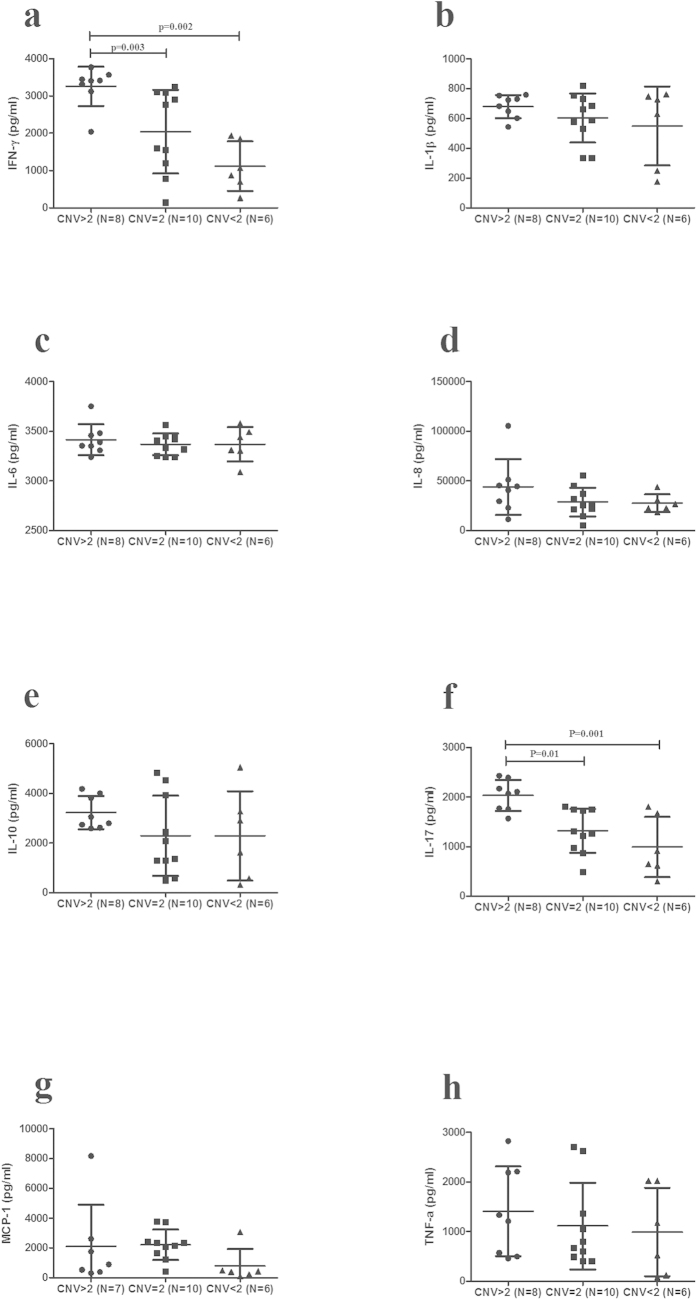
The influence of C3 CNVs on cytokines production. Different copy numbers of C3 on cytokines production. The levels of IFN-γ (**a**), IL-1β (**b**), IL-6 (**c**), IL-8 (**d**), IL-10 (**e**), IL-17 (**f**), MCP-1 (**g**), and TNF-α (**h**) in PBMCs from normal controls with different copy numbers of C3 (CNV > 2: n = 7–8, CNV = 2: n = 10, CNV < 2: n = 6). Two independent samples Nonparametric test or one-way ANOVA test was used to analyze the significance. Horizontal line represents the mean level.

**Figure 4:**
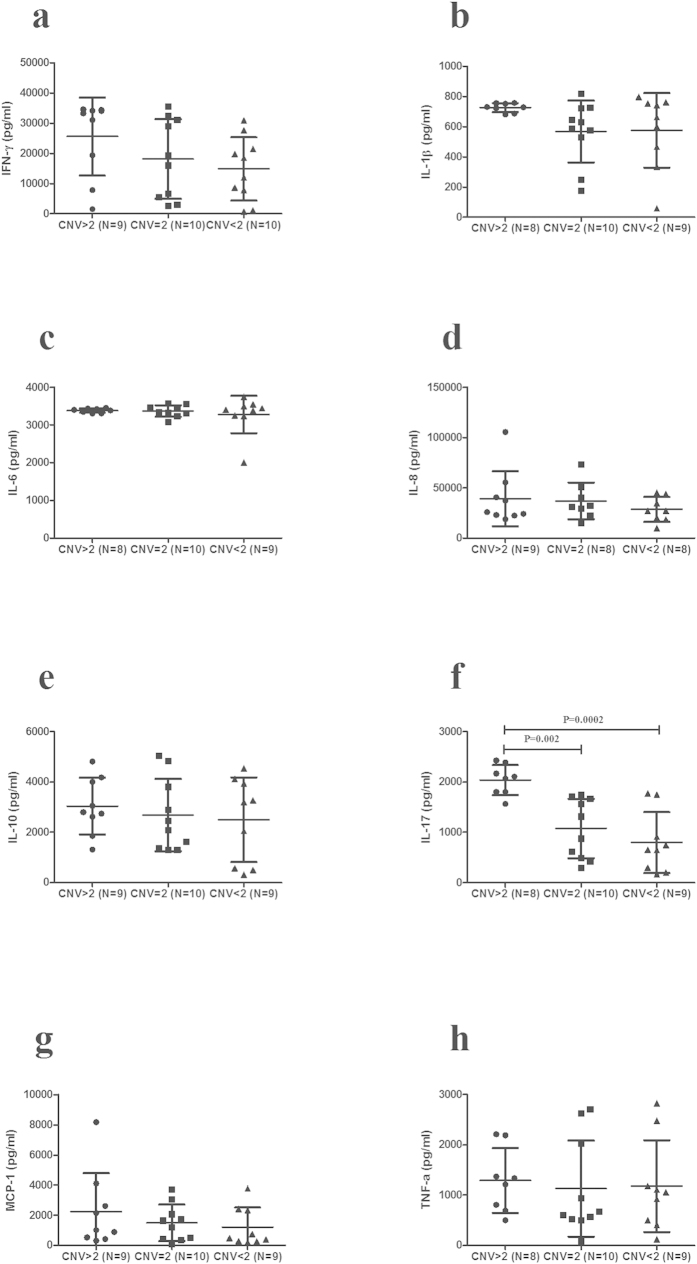
The influence of C5 CNVs on cytokines production. Different copy numbers of C5 on cytokines production. The levels of IFN-γ (**a**), IL-1β (**b**), IL-6 (**c**), IL-8 (**d**), IL-10 (**e**), IL-17 (**f**), MCP-1 (**g**), and TNF-α (**h**) in PBMCs from normal controls with different copy numbers (CNV > 2: n = 8–9, CNV = 2: n = 8–10, CNV < 2: n = 8–10). Significance was tested by two independent samples Nonparametric test or one-way ANOVA test.

A further experiment was performed to examine whether different CNVs of C3 and C5 influenced cytokine production. Culture supernatants of stimulated PBMCs from genotyped healthy controls were tested for IL-17, IFN-γ, TNF-α, IL-10, IL-1β, MCP-1, IL-6 and IL-8. The results demonstrated that IL-17 production was increased in individuals carrying more than 2 copies of the C3 gene as compared with those with less than 2 copies (P = 0.001) or 2 copies (P = 0.01). The level of IFN-γ showed a similar result as IL-17 (Fig. 3). The IL-17 produced in high C5 copy number group was significantly increased as compared to the other two groups (<2 copies (P = 0.0002) and 2 copies (P = 0.002); Fig. 4).

### The influence of C3 rs408290 and C5 rs2269067 on cytokine production

**Figure 5:**
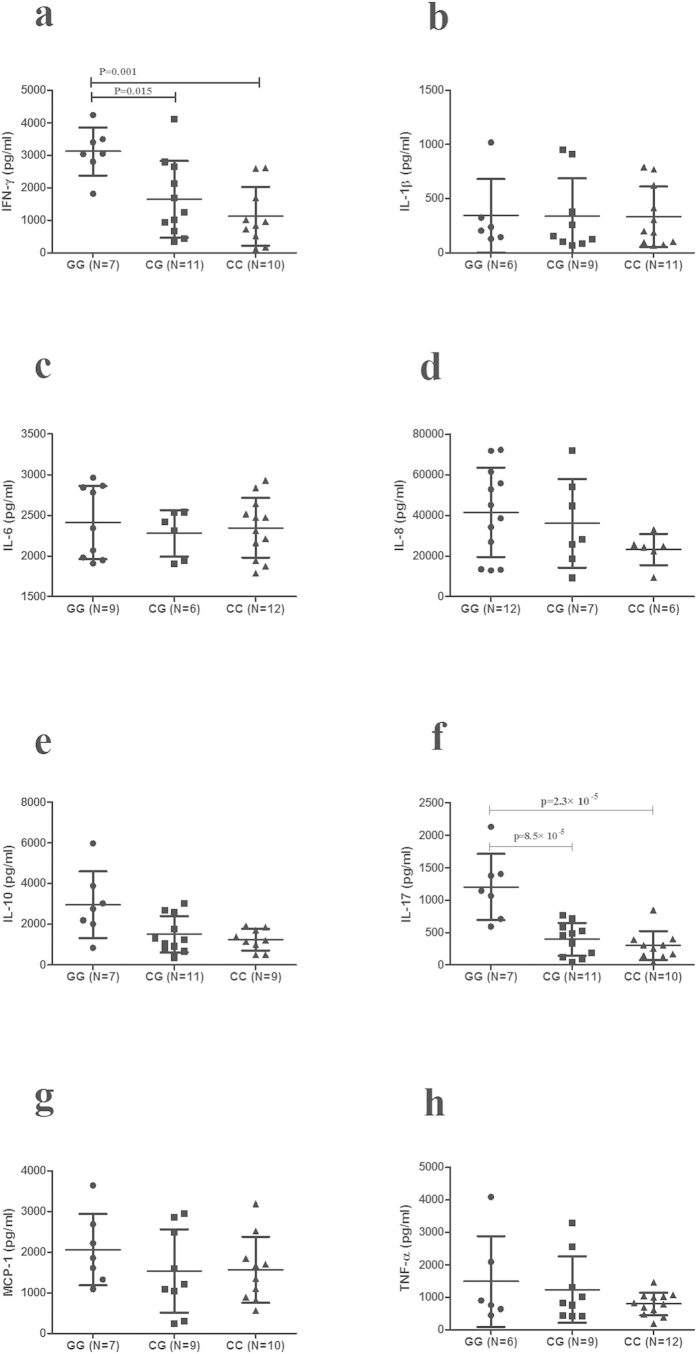
The influence of C3 rs408290 on cytokines production. Different genotypes of C3 rs408290 on cytokine production. The levels of IFN-γ (**a**), IL-1β (**b**), IL-6 (**c**), IL-8 (**d**), IL-10 (**e**), IL-17 (**f**), MCP-1 (**g**), and TNF-α (**h**), in PBMCs from normal controls with different genotypes (GG: n = 6–12, CG: n = 6–11, CC: n = 6–12). Significance was tested by two independent samples Nonparametric test or one-way ANOVA test. Data are shown as mean ± SD.

**Figure 6:**
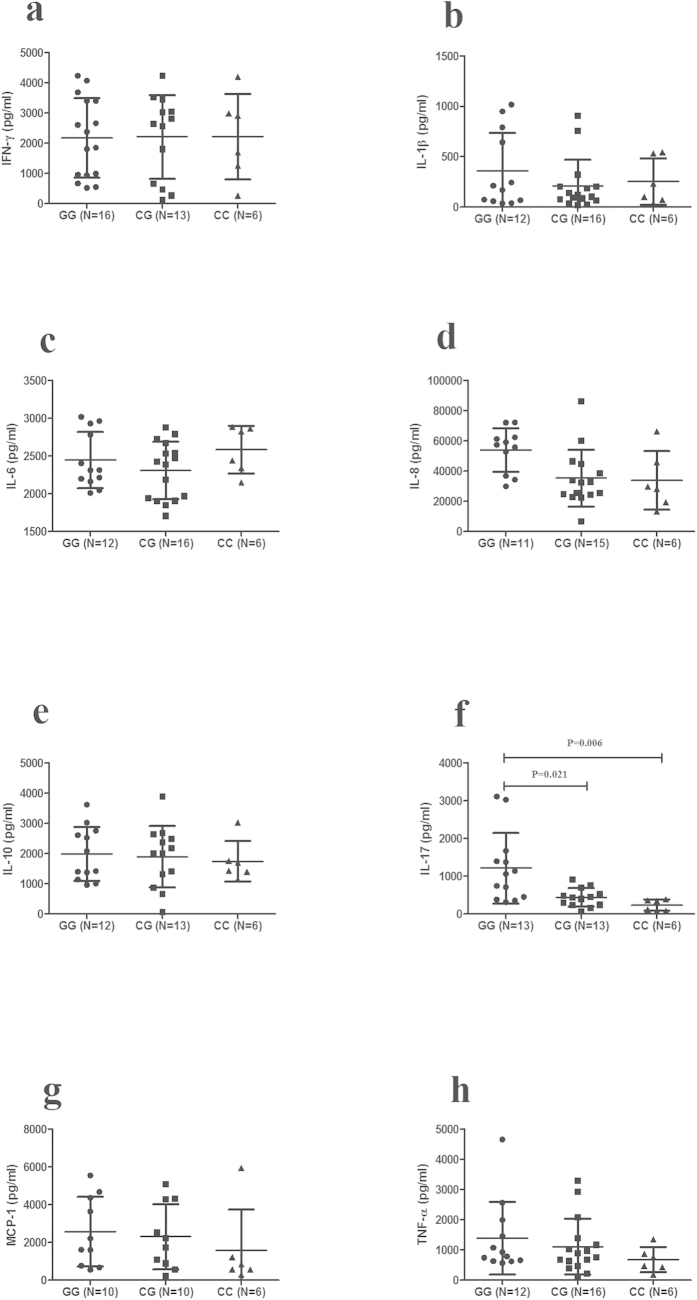
The influence of C5 rs2269067 on cytokines production. Cytokines production of C5 rs2269067 with different genotype cases. The levels of IFN-γ (**a**), IL-1β (**b**), IL-6 (**c**), IL-8 (**d**), IL-10 (**e**), IL-17 (**f**), MCP-1 (**g**), and TNF-α (**h**) in PBMCs from normal controls with different genotypes (GG: n = 10–16, CG: n = 10–16, CC: n = 6). Significance was examined by two independent samples Nonparametric test or one-way ANOVA test. Data are shown as mean ± SD.

The effect of C3 or C5 genotype on the production of cytokines by stimulated PBMCs was also investigated using genotyped healthy individuals. The results showed that the level of IL-17 in C3 rs408290 GG cases was significantly higher than in CG cases (P = 8.5 × 10^−5^) and CC cases (P = 2.3 × 10^−5^). The IFN-γ production in rs408290 GG cases was also higher than in CG cases (P = 0.015) and CC cases (P = 0.001) (Fig. 5). The production of IL-17 in C5 rs2269067 GG cases was higher than in CG genotypes and CC genotypes (P = 0.021, P = 0.006, respectively) (Fig. 6).

## Discussion

The present study shows that a high gene copy number of the complement component C3 is a risk factor for BD and VKH syndrome. A high gene copy number of complement component C5 is a risk for BD but not for VKH. Furthermore, the rs408290 GG genotype of C3 and rs2269067 GG genotype of C5 were a risk factor for BD but not for VKH syndrome. Functional studies showed that the mRNA expression in the high copy number group and GG genotype cases of C3 and C5 were all increased as compared with the other two type cases. Additionally, IL-17 and IFN-γ were significantly increased in individuals carrying more than two C3 gene copies and in those with the C3 rs408290 GG genotype. The production of IL-17 was also increased in the more than two C5 copies number group and in the C5 rs2269067 GG genotype cases.

Our selection of SNPs of the complement components was based on previous studies with autoimmune or autoinflammatory disorders^21^,^22^,^23^,^24^,^25^,^26^,^27^,^28^,^29^,^30^,^31^. Since there were no literature reports concerning the association of immune disorders with gene polymorphisms of C6, C7, and C8, we did not include these in our study. Seddon *et al.*^21^ found that rs34882957 of C9 was associated with susceptibility to age-related macular degeneration in Americans and Europeans. The SNP rs34882957 was not included in our study because it does not show variation in Asians. According to previous studies, a total of nine SNPs of C3 including rs7951, rs344555, rs2230199, rs1047286, rs171094, rs2250656, rs3745568, rs2241394 and rs147859257 were associated with autoimmune or autoinflammatory disorders^21^,^22^,^23^,^24^,^25^,^26^,^27^,^28^. Among of them, three SNPs of C3 (rs2230199, rs1047286, rs147859257) were monozygous. Two pairs of SNPs (rs3745568 and rs408290, rs171094 and rs2250656) are in linkage disequilibrium (D = 0.91, r^2^ = 0.45 and D = 1, r^2^ = 0.13, respectively). Two SNPs (rs408290 and rs432001) were selected from TagSNPs. Finally, six C3 SNPs including rs408290, rs7951, rs344555, rs2250656, rs432001, and rs2241394 were selected for our study. Ten SNPs of C5 (rs2269067, rs17611, rs7026551, rs10985126, rs7037673, rs1468673, rs7040033, rs2269066, rs25681, rs7027797) were associated with autoimmune or autoinflammatory disease in previous studies^21^,^29^,^30^,^31^. Among of them, five SNPs (rs7026551, rs2269066, rs10985126, rs1468673 and rs2269067) are in linkage disequilibrium (r^2^ ≥ 0.86). Four SNPs including rs7037673, rs17611, rs25681, rs7040033 also showed strong linkage disequilibrium (r^2^ = 1). One SNP (rs1017119) was selected from TagSNP. Finally, four SNPs of C5 including rs2269067, rs7040033, rs1017119 and rs7027797 were selected in this study. All in all, a total of ten SNPs were genotyped in our study.

To our knowledge, the possible association of C3, C5, C6, C7, C8A, C8B and C9 CNVs with autoimmune or autoinflammatory disease has not yet been reported. Our study is the first to identify the association of C3 CNVs with BD and VKH syndrome, and the association of C5 CNVs with BD. We also examined the association of C6, C7, C8A, C8B and C9 CNVs with BD and VKH syndrome, but no association could be detected.

Additionally, we identified the association of C3 polymorphisms with BD. Previous studies showed that the genetic polymorphisms of C3 are associated with immune-related diseases. Miyagawa *et al.*^22^ reported that a polymorphism of the C3 gene was associated with SLE. Yates *et al.*^24^ found an association between C3 polymorphisms and age-related macular degeneration. Additionally, Ghannam *et al.*^32^ reported that T cells from C3 deficient patients could not produce IFN-γ, suggesting that C3 plays a critical role in the production of IFN-γ. Sugihara *et al.*^33^ reported that C3 could promote IL-17 release by NF-κB activation. The present study showed that the C3 rs408290 SNP was associated with susceptibility to acquire BD. The mRNA expression and cytokine production including IL-17 and IFN-γ of individuals carrying the C3 rs408290 GG genotype was increased. These findings support the hypothesis that the complement component C3 may play an important role in the pathogenesis of BD or VKH syndrome through an enhanced production of IL-17 and IFN-γ. These findings confirm earlier data from our group showing the increased expression of these two cytokines in VKH and BD^34^,^35^.

C5 plays an important role during innate immunity whereby the generation of C5a leads to a powerful leukocyte chemotaxis, but may also modulate the immune response^36^. Many studies have shown that polymorphisms of C5 are associated with autoimmune or autoinflammatory disorders. Chang *et al.*^29^ reported that SNP rs2269067 of C5 showed a strong association with RA. Additionally, experiments showed that C5a blockade could reduce the progression and severity of experimental autoimmune uveitis^20^ and experimental arthritis^37^ via a reduced production of IFN-γ. Lajoie *et al.*^38^ found that C5a could regulate Th17 differentiation and proliferation to reduce the production of IL-17A by inhibiting IL-23 expression. These studies suggested that C5 plays an important role in immune-related disorders via enhanced production of IFN-γ and IL-17A. The present data from CNVs and SNPs demonstrated that C5 was a risk for BD. The mRNA expression was enhanced in the pathogenic type cases of C5 (high copy number cases and rs2269067 GG genotype cases). Functional studies showed increased IL-17 production in the high copy number group and in GG genotype cases. Consistent with previous studies, the present findings suggested that C5 may play a role in the onset of BD by increasing the production of IL-17.

In our study we found that C3 and C5 CNVs and SNPs had a significant effect on important cytokine markers of the Th1 (IFN-γ) and Th17 (IL-17) response. On the other hand we were not able to show an effect on other cytokines such as IL-1β, IL-6, IL-8, IL-10, MCP-1 or TNF-α. Although the approach we chose (LPS stimulation of genotype PBMCs) did not reveal an effect on proinflammatory cytokine production we cannot exclude that the various C3 and C5 genetic variants may affect proinflammatory cytokine production via other pathways. Our method to study the effect of C3 and C5 CNVs and SNPs by antigen or LPS stimulated PBMCs has not yet been reported in the literature and further studies are needed to confirm the effect of these genotypes on the inflammatory response.

Our SNP study in BD and VKH syndrome showed that C3 rs408290 and C5 rs2269067 were associated with BD risk, but we did not find an association for the ten SNPs tested with VKH syndrome. The discrepancy in complement component polymorphisms between BD and VKH syndrome may be due to the fact that different immune mediated pathways are operative in these two uveitis entities. VKH is now seen as an autoimmune disorder, whereas BD is considered as an autoinflammatory disease^39^,^40^.

There are several limitations in our study that should be considered. Firstly, the power calculation showed that the sample size was large enough to examine the difference between cases and controls for the positive association of CNVs and SNPs. However, a larger sample size is needed to prove the negative association of C6 and C7 CNVs or rs2241394/C3, rs7027797/C5 and rs1017119/C5 genotypes (Supplementary Tables 12–13). Secondly, the participants recruited from our ophthalmology department may cause a selective bias for BD. The results of a stratified analysis however did not show an association of copy number variations of C3 and C5 and genotypes of rs408290 and rs2269067 with the main clinical features such as arthritis and skin lesions of BD. However, our results should be confirmed by a collaboration with other departments seeing BD patients, such as dermatology and rheumatology. Thirdly, the patients selected all belonged to a Chinese Han population and our results therefore need to be confirmed in other ethnic populations. Finally, the functional studies were performed in healthy genotyped individuals and not in patients, to exclude a confounding effect of the inflammatory response or installed therapy. Future studies could be planned to investigate how disease activity affects the mRNA expression and cytokine levels in genotyped patients.

In conclusion, the results revealed that a high gene copy number of C3 is a risk factor for BD and VKH syndrome, and that more than two copies of C5 was a risk for BD. The C3 rs408290 and C5 rs2269067 SNPs showed a strong association with BD. Moreover our study provides evidence for an involvement of C3 and C5 genes in BD or VKH syndrome in the modulation of Th1 and Th17 cytokine production.

## Methods

### Clinical Samples

The study objects contained 1064 patients with BD, 1059 patients with VKH syndrome and 2174 normal controls. All the participants were enrolled from the First Affiliated Hospital of Chongqing Medical University (Chongqing, China) and the Zhongshan Ophthalmic Center of Sun Yat-sen University (Guangzhou, China). BD and VKH syndrome were strictly diagnosed based on the criteria of the International Study Group for Behcet’s disease and First International Workshop for VKH syndrome^41^,^42^, respectively. The patients were excluded if the diagnosis was in doubt.

### Ethics Statement

All venous blood samples were collected after approval from the relevant research ethics committees and written informed consent was obtained from all the participants. All procedures in this study were based on the principles of the Declaration of Helsinki. The study was approved by the Clinical Research Ethics Committee of the First Affiliated Hospital of Chongqing Medical University (Permit Number: 2009-201008). All methods were carried out in accordance with the approved guidelines.

### DNA extraction and copy number variations analysis

Genomic DNA was extracted from peripheral blood in patients and controls using Qiagen QIAamp DNA Mini blood kit (Qiagen, Valencia, California, USA). The CNVs of C3 (Hs00264021-cn), C5 (Hs00129394-cn), C6 (Hs03574047-cn), C7 (Hs03555097-cn), C8A (Hs01058812-cn), C8B (Hs05747231-cn) and C9 (Hs02856091-cn) was detected by real-time PCR and repeated three times for each sample using TaqMan assays labeled with FAM (Applied Biosystems, Foster City, California, USA). TaqMan RNase P assay labeled with VIC was used as the internal copy number reference (Applied Biosystems, Foster City, California, USA).

### SNP genotyping

Among the six SNPs of C3 and four SNPs of C5, eight SNPs including rs2269067, rs7040033, rs1017119 rs7027797, rs408290, rs7951, rs344555, and rs2250656 were genotyped by PCR-RFLP method. Two SNPs including rs432001 and rs2241394 were genotyped by PCR assay (TaqMan assay ID: C_987 748_1, C_15873105_10, respectively) (Applied Biosystems, Foster City, California, USA) on real-time PCR (Applied Biosystems, Foster City, California, USA). The proper primers were contributed to the target DNA sequence amplification by PCR. Restriction enzymes were used to digest the PCR products (The primer sequences and restriction enzymes are shown in Supplementary Table 1). Four percent agarose gels were applied to separate the digestion products and colored with GoldView (SBS Genetech, Beijing, China). In order to assure the balance between patients and controls, Hardy-Weinberg equilibrium was applied to analyze the frequencies of genotype and allele. Direct sequencing was applied to 10% of study samples in a random fashion to assure the validity of the SNP genotyping method used.

### Cell isolation and culture

PBMCs were isolated by using Ficoll-Hypaque density-gradient centrifugation, and then seeded in 24-well plates (2 × 106 cells per well) and cultured for 1 day or 3 days. The culture system comprised 100 μg/ml streptomycin, RPMI medium 1640 supplemented with 10% fetal calf serum (FCS, Greiner, Wemmel, Belgium), 100U/ml penicillin (Invitrogen, Carlsbad, USA). PBMCs were stimulated with a mixture of anti-CD3 (5 μg/ml, eBioscience, SanDiego, California, USA) and anti-CD28 antibodies (1 μg/ml, eBioscience, SanDiego, California, USA) for 72 hours where after IL-17, IFN-γ, and IL-10 levels were measured in the culture supernatants. To detect the production of IL-1β, IL-8, TNF-α, IL-6, and MCP-1, PBMCs were cultured with 100 ng/ml lipopolysaccharide (100 ng/ml, Sigma, Missouri, USA) for 24 hours.

### Real-time PCR

TRIzol (Invitrogen, San Diego, California, USA) and transcriptase kit (Applied Biosystems, Foster City, California, USA) were used for total RNA extraction and cDNA reverse transcription, respectively. The primer sequences for C3 and C5 were as follows: forward: 5′-GGAGCAGTCAAGGTCTAC GC-3′, reverse: 5′-CACAGTTCATCACGGCAG AG-3′; forward: 5′-GGCACAAA GTCCTCCAAATG-3′, reverse: 5′-CCAAACCA AGTCTCCAGTGA-3′, respectively. β-actin was selected as the internal reference gene and the 2^−ΔΔCt^ method was used to calculate the relative expression for each gene.

### ELISA

The production of IL-17, IFN-γ, IL-10, IL-8, TNF-α, IL-6, IL-1β and MCP-1 from PBMC culture supernatants was detected by using the human Duoset ELISA development kit (R&D Systems, Minneapolis, Minnesota, USA) based on the manufacturer’s protocols.

### Statistical analysis

Two steps were applied to analyze the data of CNVs. Data from Real-time PCR was first analyzed to accept Ct results by using 7500 system software, version 2.0.6 (Applied Biosystems, Foster City, California, USA), then the comparative Ct method was used to confirm relative gene copy numbers by CopyCaller software, version 2.0 (Applied Biosystems, Foster City, California, USA). To compare the differences between patients and controls, χ2 test and chi-square test (SPSS version 17.0, Inc., Chicago, Illinois, USA) were applied to analyze the frequencies of CNVs, genotypes and alleles. The mRNA expression and various cytokines were analyzed by one-way ANOVA or a nonparametric test for independent samples. Bonferroni correction was applied to correct for multiple comparisons (SPSS version 17.0, Inc., Chicago, Illinois, USA).

## Additional Information

**How to cite this article**: Xu, D. *et al.* Copy number variations and gene polymorphisms of Complement components in ocular Behcet’s disease and Vogt-Koyanagi-Harada syndrome. *Sci. Rep.* **5**, 12989; doi: 10.1038/srep12989 (2015).

## Supplementary Material

Supplementary Information

## Figures and Tables

**Table 1:** Comparison of gene copy numbers of C3 and C5 in patients with BD, patients with VKH, and normal controls.

Gene	Stage	Copy number	BD	VKH	Control	P value of BD	Pc value of BD	OR(95%CI)	P value of VKH	Pc value of VKH	OR(95%CI)
C3	First	<2	8(0.02)	9(0.02)	10(0.02)	0.698	NS	1.2 (0.4–3.0)	0.512	NS	1.3 (0.5–3.3)
		=2	348(0.91)	350(0.91)	551(0.96)	0.001	0.021	0.4 (0.2–0.7)	0.002	0.042	0.4 (0.2–0.7)
		>2	26(0.07)	24(0.07)	12(0.02)	2.6 × 10^−4^	5.5 × 10^−3^	3.4 (1.7–6.8)	8.9 × 10^−4^	0.018	3.1 (1.5–6.3)
	Replication	<2	15(0.02)	24(0.04)	29(0.02)	0.537	NS	1.2 (0.6–2.3)	0.012	NS	1.9 (1.1–3.4)
		=2	618(0.91)	605(0.89)	1531(0.95)	3.1 × 10^−6^	6.5 × 10^−5^	0.4(0.3–0.6)	2.9 × 10^−8^	6.1 × 10^−7^	0.3 (0.2–0.5)
		>2	49(0.07)	47(0.07)	41(0.03)	2.0 × 10^−7^	4.2 × 10^−6^	2.9(1.9–4.5)	6.7 × 10^−7^	1.4 × 10^−5^	2.8 (1.8–4.3)
	Combined	<2	23(0.02)	33(0.03)	39(0.02)	0.473	NS	1.2 (0.7–2.0)	0.017	0.357	1.76 (1.1–2.8)
		=2	966(0.91)	955(0.90)	2082(0.95)	1.5 × 10^−8^	3.1 × 10^−7^	0.4(0.3–0.5)	4.1 × 10^−10^	8.6 × 10^−9^	0.4 (0.3–0.5)
		>2	75(0.07)	71(0.07)	53(0.03)	2.5 × 10^−10^	5.3 × 10^−9^	3.0(2.11–4.3)	3.0 × 10^−9^	6.4 × 10^−8^	2.8 (1.9–4.1)
C5	First	<2	7(0.02)	6(0.02)	5(0.01)	0.194	NS	2.1 (0.6–6.7)	0.327	NS	1.8 (0.5–5.9)
		=2	360(0.94)	368(0.96)	563(0.98)	1.6 × 10^−4^	3.4 × 10^−3^	0.2 (0.1–0.5)	0.013	NS	0.3 (0.1–0.8)
		>2	15(0.04)	9(0.02)	3(0.01)	1.5 × 10^−5^	3.3 × 10^−4^	7.7(2.2–26.9)	0.017	NS	4.6 (1.2–16.9)
	Replication	<2	21(0.03)		30(0.02)	0.069	NS	1.6(0.9–2.9)			
		=2	604(0.90)		1525(0.96)	4.6 × 10^−8^	9.8 × 10^−7^	0.3(0.2–0.5)			
		>2	46(0.07)		35(0.02)	5.3 × 10^−6^	1.1 × 10^−4^	3.2(2.0–5.1)			
	Combined	<2	28(0.03)		35(0.01)	0.046	NS	1.6(1.0–2.7)			
		=2	964(0.92)		2088(0.97)	6.7 × 10^−10^	1.4 × 10^−8^	0.3(0.2–0.5)			
		>2	61(0.05)		38(0.02)	5.2 × 10^−10^	1.1 × 10^−8^	3.4(2.2–5.1)			

BD, Behcet’s disease; VKH, Vogt-Koyanagi-Harada syndrome. OR, odds ratio; CI, confidence interval; Pc, P Bonferroni correction. NS, no significant different.

**Table 2:** Frequencies of genotypes and alleles of C3 and C5 in patients with BD, patients with VKH, and normal controls.

SNP	Stage	Allele	BD	VKH	Control	P^a^	Pc^a^	OR(95%CI)	P^b^	Pc^b^	OR(95%CI)
rs408290(C3)	First	C	488(0.649)	515(0.690)	782(0.696)	0.034	NS	0.80(0.66–0.98)	0.805	NS	0.97(0.79–1.19).
		G	264(0.351)	515(0.690)	342(0.304)	0.034	NS	1.23(1.01–1.50)	0.805	NS	1.02(0.83–1.25)
		CC	173(0.460)	186(0.499)	268(0.477)	0.614	NS	0.93(0.72–1.21)	0.514	NS	1.09(0.84–1.41)
		CG	142(0.378)	143(0.383)	246(0.438)	0.067	NS	0.78(0.59–1.01)	0.099	NS	0.79(0.61–1.04)
		GG	61(0.162)	44(0.118)	48(0.085)	3.2 × 10^−4^	8.6 × 10^−3^	2.07(1.38–3.10)	0.102	NS	1.43(0.93–2.20)
	Replication	C	905(0.791)		1279(0.792)	0.931	NS	0.99(0.82–1.19)			
		G	239(0.209)		335(0.208)	0.931	NS	1.01(0.83–1.21)			
		CC	380(0.664)		496(0.615)	0.059	NS	1.24(0.99–1.55)			
		CG	145(0.253)		287(0.356)	5.6 × 10^−5^	1.5 × 10^−3^	0.61(0.48–0.78)			
		GG	47(0.082)		24(0.030)	1.4 × 10^−5^	3.7 × 10^−4^	2.92(1.76–4.83)			
	Combined	C	1393(0.735)		2061(0.753)	0.166	NS	0.91(0.79–1.04)			
		G	503(0.265)		677(0.247)	0.166	NS	1.09(0.96–1.25)			
		CC	553(0.583)		764(0.558)	0.227	NS	1.10(0.93–1.31)			
		CG	287(0.303)		533(0.389)	1.8 × 10^−5^	4.9 × 10^−4^	0.68(0.57–0.81)			
		GG	108(0.114)		72(0.053)	5.8 × 10^−8^	1.5 × 10^−7^	2.31(1.69–3.16)			
rs2269067(C5)	First	C	125(0.169)	183(0.235)	274(0.245)	1.1 × 10^−4^	1.1 × 10^−3^	0.63(0.49–0.79)	0.637	NS	0.95(0.76–1.17)
		G	613(0.831)	595(0.765)	846(0.755)	1.1 × 10^−4^	1.1 × 10^−3^	1.58(1.25–2.01)	0.637	NS	1.05(0.85–1.30)
		CC	15(0.041)	22(0.057)	28(0.050)	0.507	NS	0.80(0.42–1.52)	0.657	NS	1.13(0.64–2.02)
		CG	95(0.257)	139(0.357)	218(0.389)	3.2 × 10^−5^	8.6 × 10^−4^	0.54(0.40–0.72)	0.318	NS	0.87(0.66–1.14)
		GG	259(0.702)	228(0.586)	314(0.561)	1.5 × 10^−5^	4.1 × 10^−4^	1.84(1.39–2.43)	0.437	NS	1.10(0.85–1.44)
	Replication	C	218(0.178)		368(0.244)	3.0 × 10^−5^	3.0 × 10^−4^	0.67(0.55–0.81)			
		G	1004(0.822)		1138(0.756)	3.0 × 10^−5^	3.0 × 10^−4^	1.48(1.23–1.79)			
		CC	29(0.047)		46(0.061)	0.269	NS	0.76(0.47–1.23)			
		CG	160(0.262)		276(0.367)	3.7 × 10^−5^	9.9 × 10^−4^	0.61(0.48–0.77)			
		GG	422(0.691)		431(0.572)	3.1 × 10^−6^	8.4 × 10^−5^	1.66(1.33–2.08)			
	Combined	C	343(0.175)		642(0.244)	1.4 × 10^−8^	1.4 × 10^−7^	0.65(0.56–0.75)			
		G	1617(0.825)		1984(0.756)	1.4 × 10^−8^	1.4 × 10^−7^	1.52(1.31–1.76)			
		CC	44(0.045)		74(0.056)	0.219	NS	0.78(0.53–1.15)			
		CG	255(0.260)		494(0.376)	4.6 × 10^−9^	1.2 × 10^−7^	0.58(0.48–0.69)			
		GG	681(0.695)		745(0.567)	4.7 × 10^−10^	1.3 × 10^−8^	1.73(1.45–2.06)			

P^a^, P value of BD. Pc^a^, Bonferroni corrected p value of BD. P^b^, P value of VKH. Pc^b^, Bonferroni corrected p value of VKH.
