# Supplementary material for: Copy number variations and gene polymorphisms of Complement components in ocular Behcet’s disease and Vogt-Koyanagi-Harada syndrome
Source: Sci Rep. 2015 Aug 13;5:12989. doi: 10.1038/srep12989 (PMC4534762; doi:10.1038/srep12989)
Supplement: Supplementary Information [file srep12989-s1.doc]

# Copy number variations and gene polymorphisms of Complement components in ocular Behcet’s disease and Vogt-Koyanagi-Harada syndrome

Dengfeng Xu^1,3^, Shengping Hou^1,3^, Jun Zhang^1^, Yanni Jiang^1^, Aize Kijlstra^2^, Peizeng Yang^1^

^1^The First Affiliated Hospital of Chongqing Medical University, Chongqing Key Laboratory of Ophthalmology and Chongqing Eye Institute, Chongqing, P. R. China

^2^University Eye Clinic Maastricht, Maastricht, The Netherlands

^3^These authors contributed equally to this work

Correspondence to: Professor Peizeng Yang, MD, PhD.

The First Affiliated Hospital of Chongqing Medical University, Chongqing Key Laboratory of Ophthalmology and Chongqing Eye Institute, Chongqing, P. R. China, 400016;

Phone: +8623 89012851;

FAX: +8623 89012851;

Email: peizengycmu@126.com

Supplementary Files

Supplementary Table 1 Primers and restriction enzymes used for restricted fragment length polymorphisms analysis of the C3 and C5 genes.

| **Gene** | **SNPs** | **Primer** | **Restriction enzyme** |
| --- | --- | --- | --- |
| **C3** | rs7951 | 5’ gggagaccaggatgccactat 3’  5’ gctccttcccctacaactcag 3’ | HindIII |
|  | rs408290 | 5’CTTGCTATGTGACTCTTGGCTGAAT 3’  5’TCGGACAAGAAAGGTGAGAGAGGAT 3’ | BtgI |
|  | rs2250656 | 5’ gcccctcctcctataagctct 3’  5’ gggcattgtcaggggtct 3’ | Hpy188I |
|  | rs344555 | 5’ AGCCACACTTACCCAGGAC 3’  5’ CCGTTCTTCCAGGGTGAC 3’ | MaeII |
| **C5** | rs2269067 | 5’ ggcccctctgtacttccatgt 3’  5’ gccagtagaggtaaatgaagcac 3’ | DraIII-HF |
|  | rs7040033 | 5’ cacacatggaaatcaagtaac 3’  5’ aaattcacttcagtaaacaggta 3’ | Acc65I |
|  | rs1017119 | 5’ ccgccttctgggttcaa 3’  5’ aagcataagattgtaccgtttt 3’ | NspI |
|  | rs7027797 | 5’ AGGGCAGGGAGAGGAATACAA 3’  5’ TGCCCTCTTCAATGTCTCTTTCA 3’ | FokI |

Supplementary Table 2 Clinical characteristic, age and gender distribution for SNPs in patients with BD and patients with VKH

| **Extraocular findings** | **Total** | **%** |
| --- | --- | --- |
| **Patients with BD**  **for SNP analysis** | 980 |  |
| Mean age±SD | 33.6±9.0 |  |
| male | 818 | 83.4 |
| female | 162 | 16.6 |
| uveitis | 980 | 100 |
| Oral ulcer | 980 | 100 |
| Genital ulcer | 559 | 57.0 |
| Skin lesions | 436 | 44.5 |
| Arthritis | 140 | 14.3 |
| Positive pathergy test | 264 | 26.9 |
| **Patients with VKH**  **for SNP analysis** | 379 |  |
| Mean age±SD | 37.7±13.2 |  |
| male | 209 | 55.1 |
| female | 170 | 44.9 |
| uveitis | 379 | 100 |
| Headache | 57 | 15.0 |
| Tinnitus | 26 | 6.86 |
| Vitiligo | 4 | 1.05 |
| Alopecia and poliosis | 3 | 0.79 |
| **controls for SNP analysis** | 1368 |  |
| Mean age±SD | 39.4±10.6 |  |
| male | 776 | 56.7 |
| female | 592 | 43.3 |

Supplementary Table 3 Clinical characteristic, age and gender distribution for CNVs in patients with BD and patients with VKH

| **Extraocular featrures** | **Total** | **%** |
| --- | --- | --- |
| **Patients with BD**  **for CNV analysis** | 1064 |  |
| Mean age±SD | 33.7±9.0 |  |
| male | 885 | 83.2 |
| female | 179 | 16.8 |
| uveitis | 1064 | 100 |
| Oral ulcer | 1064 | 100 |
| Genital ulcer | 489 | 45.9 |
| Skin lesions | 379 | 35.6 |
| Arthritis | 164 | 15.4 |
| Positive pathergy test | 283 | 25.6 |
| **Patients with VKH**  **for CNV analysis** | 1059 |  |
| Mean age±SD | 39.6±13.9 |  |
| male | 582 | 54.9 |
| female | 477 | 45.1 |
| uveitis | 1059 | 100 |
| Headache | 224 | 21.1 |
| Tinnitus | 132 | 12.5 |
| Vitiligo | 28 | 2.64 |
| Alopecia and poliosis | 16 | 1.51 |
| **controls for CNV analysis** | 2174 |  |
| Mean age±SD | 39.6±10.7 |  |
| male | 1218 | 56.0 |
| female | 956 | 44.0 |

Supplementary Table 4 CNVs analysis of C6, C7, C8A, C8B, C9 in patients with BD, patients with VKH, and normal controls

| Gene | Copy  number | BD | VKH | Control | P value  of BD | Pc value  of BD | OR(95%CI) | P value  of VKH | Pc value  of VKH | OR(95%CI) |
| --- | --- | --- | --- | --- | --- | --- | --- | --- | --- | --- |
| C6 | <2 | 30(0.08) | 19(0.05) | 52(0.10) | 0.49 | NS | 0.8(0.5-1.4) | 0.011 | NS | 0.5(0.3-0.9) |
|  | =2 | 314(0.85) | 314(0.82) | 439(0.79) | 0.04 | NS | 1.4(1.0-2.1) | 0.351 | NS | 1.2(0.8-1.6) |
|  | >2 | 26(0.07) | 50(0.13) | 61(0.11) | 0.04 | NS | 0.6(0.4-1.0) | 0.351 | NS | 1.2(0.8-1.8) |
| C7 | <2 | 14(0.04) | 12(0.03) | 20(0.04) | 0.85 | NS | 1.0(0.5-2.1) | 0.704 | NS | 0.9(0.4-1.8) |
|  | =2 | 334(0.89) | 345(0.93) | 526(0.94) | 0.09 | NS | 0.6(0.4-0.9) | 0.557 | NS | 1.2(0.7-2.2) |
|  | >2 | 28(0.07) | 16(0.04) | 15(0.02) | 0.03 | NS | 2.1(1.0-4.1) | 0.644 | NS | 0.8(0.3-2.2) |
| C8A | <2 | 11(0.03) | 12(0.03) | 8(0.01) | 0.11 | NS | 2.1(0.8-5.3) | 0.080 | NS | 2.0(0.9-5.4) |
|  | =2 | 332(0.91) | 338(0.91) | 538(0.96) | 0.04 | NS | 0.3(0.1-0.5) | 0.030 | NS | 0.2(0.1-0.4) |
|  | >2 | 23(0.06) | 21(0.06) | 16(0.03) | 0.01 | NS | 6.1(2.5-15.3) | 0.020 | NS | 8.6(3.6-20.7) |
| C8B | <2 | 19(0.05) | 15(0.04) | 12(0.02) | 0.012 | NS | 2.5(1.2-5.2) | 0.097 | NS | 1.9(0.9-4.1) |
|  | =2 | 338(0.90) | 347(0.91) | 543(0.95) | 0.003 | NS | 0.5(0.2-0.8) | 0.007 | NS | 0.5(0.2-0.8) |
|  | >2 | 18(0.05) | 20(0.05) | 15(0.03) | 0.08 | NS | 1.9(0.9-3.7) | 0.036 | NS | 2.0(1.0-4.0) |
| C9 | <2 | 27(0.07) | 25(0.06) | 22(0.04) | 0.028 | NS | 1.9(1.1-3.4) | 0.052 | NS | 1.8(0.9-3.2) |
|  | =2 | 335(0.88) | 332(0.89) | 532(0.93) | 0.003 | NS | 0.5(0.3-0.8) | 0.010 | NS | 0.5(0.3-0.8) |
|  | >2 | 20(0.05) | 18(0.05) | 16(0.03) | 0.054 | NS | 1.9(0.9-3.7) | 0.108 | NS | 1.7(0.8-3.4) |

BD, Behcet’s disease. VKH, Vogt–Koyanagi–Harada syndrome. OR, odds ratio. CI, confidence interval. Pc, P Bonferroni correction. NS, no significant different

Supplementary Table 5 Frequencies of genotypes and alleles of complement C3 polymorphisms in patients with BD, patients with VKH, and normal controls

| Genes | SNPs | Allele | BD | Control | P value | Pc value | OR(95%CI) | VKH | Control | P value | Pc value | OR(95%CI) |
| --- | --- | --- | --- | --- | --- | --- | --- | --- | --- | --- | --- | --- |
| C3 | rs7951 | T | 72(0.094) | 104(0.092) | 0.848 | NS | 1.03(0.75-1.41) | 68(0.091) | 104(0.092) | 0.929 | NS | 0.98(0.71-1.35) |
|  |  | C | 690(0.906) | 1028(0.908) | 0.848 | NS | 0.97(0.70-1.32) | 682(0.909) | 1028(0.908) | 0.929 | NS | 1.01(0.73-1.39) |
|  |  | CC | 315(0.826) | 465(0.822) | 0.836 | NS | 1.04(0.73-1.46) | 310(0.826) | 465(0.822) | 0.840 | NS | 1.03(0.73-1.46) |
|  |  | CT | 60(0.157) | 98(0.173) | 0.526 | NS | 0.89(0.62-1.26) | 62(0.165) | 98(0.173) | 0.755 | NS | 0.94(0.66-1.34) |
|  |  | TT | 6(0.017) | 30(0.005) | 0.104 | NS | 3.00(0.74-12.08) | 3(0.009) | 30(0.005) | 0.610 | NS | 1.51(0.30-7.53) |
|  | rs2250656 | A | 626(0.823) | 888(0.775) | 0.010 | NS | 1.35(1.07-1.71) | 563(0.742) | 888(0.775) | 0.107 | NS | 0.83(0.67-1.03) |
|  |  | G | 134(0.177) | 258(0.225) | 0.010 | NS | 0.73(0.58-0.93) | 195(0.258) | 258(0.225) | 0.107 | NS | 1.19(0.96-1.47) |
|  |  | AA | 249(0.655) | 326(0.569) | 0.008 | NS | 1.44(1.10-1.88) | 189(0.498) | 326(0.569) | 0.033 | NS | 0.75(0.58-0.97) |
|  |  | AG | 128(0.336) | 236(0.412) | 0.02 | NS | 0.72(0.55-0.95) | 185(0.488) | 236(0.412) | 0.020 | NS | 1.36(1.04-1.76) |
|  |  | GG | 3(0.009) | 11(0.019) | 0.15 | NS | 0.41(0.11-1.46) | 5(0.014) | 11(0.019) | 0.480 | NS | 0.68(0.23-1.98) |
|  | rs432001 | A | 669(0.906) | 1027(0.923) | 0.193 | NS | 0.80(0.57-1.12) | 670(0.888) | 1027(0.923) | 0.010 | NS | 0.66(0.48-0.91) |
|  |  | G | 69(0.094) | 85(0.077) | 0.193 | NS | 1.24(0.89-1.74) | 84(0.112) | 85(0.077) | 0.010 | NS | 1.51(1.10-2.08) |
|  |  | AA | 300(0.813) | 465(0.836) | 0.173 | NS | 0.78(0.55-1.11) | 293(0.777) | 465(0.836) | 0.006 | NS | 0.63(0.45-0.88) |
|  |  | AG | 69(0.187) | 91(0.164) | 0.173 | NS | 1.27(0.89-1.81) | 84(0.223) | 91(0.164) | 0.006 | NS | 1.59(1.13-2.22) |
|  | rs344555 | G | 527(0.699) | 829(0.731) | 0.129 | NS | 0.85(0.69-1.04) | 509(0.686) | 829(0.731) | 0.035 | NS | 0.80(0.65-0.98) |
|  |  | A | 227(0.301) | 305(0.269) | 0.129 | NS | 1.17(0.95-1.43) | 233(0.314) | 305(0.269) | 0.035 | NS | 1.24(1.01-1.52) |
|  |  | AA | 26(0.069) | 43(0.075) | 0.691 | NS | 0.90(0.54-1.49) | 40(0.107) | 43(0.075) | 0.092 | NS | 1.47(0.93-2.31) |
|  |  | AG | 175(0.464) | 219(0.386) | 0.017 | NS | 1.37(1.05-1.79) | 153(0.412) | 219(0.386) | 0.423 | NS | 1.11(0.85-1.45) |
|  |  | GG | 176(0.467) | 305(0.539) | 0.032 | NS | 0.75(0.57-0.97) | 178(0.481) | 305(0.539) | 0.082 | NS | 0.79(0.61-1.03) |
|  | rs2241394 | C | 691(0.936) | 1029(0.930) | 0.618 | NS | 1.10(0.75-1.60) | 699(0.927) | 1029(0.930) | 0.784 | NS | 0.95(0.66-1.36) |
|  |  | G | 47(0.064) | 77(0.070) | 0.618 | NS | 0.91(0.62-1.32) | 55(0.073) | 77(0.070) | 0.784 | NS | 1.05(0.73-1.50) |
|  |  | CC | 322 (0.872) | 476 (0.861) | 0.605 | NS | 1.10(0.75-1.63) | 322 (0.854) | 476 (0.861) | 0.775 | NS | 0.94(0.65-1.37) |
|  |  | GG | 47(0.128) | 77(0.139) | 0.605 | NS | 0.90(0.61-1.33) | 55(0.146) | 77(0.139) | 0.775 | NS | 1.05(0.72-1.53) |

Pc, P Bonferroni correction. OR, odds ratio. CI, confidence interval. NS, no significant different

Supplementary Table 6 Frequencies of genotypes and alleles of complement C5 polymorphisms in patients with BD, patients with VKH, and normal controls

| Genes | SNPs | Allele | BD | Control | P value | Pc value | OR(95%CI) | VKH | Control | P value | Pc value | OR(95%CI) |
| --- | --- | --- | --- | --- | --- | --- | --- | --- | --- | --- | --- | --- |
| C5 | rs7040033 | A | 429(0.579) | 662(0.579) | 0.998 | NS | 1.00(0.82-1.20) | 438(0.590) | 662(0.579) | 0.648 | NS | 1.04(0.86-1.26) |
|  |  | G | 311(0.421) | 480(0.421) | 0.998 | NS | 1.00(0.82-1.20) | 304(0.410) | 480(0.421) | 0.648 | NS | 0.95(0.79-1.15) |
|  |  | GG | 45(0.121) | 90(0.157) | 0.124 | NS | 0.74(0.50-1.08) | 40(0.108) | 90(0.157) | 0.03 | NS | 0.64(0.43-0.96) |
|  |  | AG | 221(0.597) | 300(0.525) | 0.03 | NS | 1.34(1.02-1.74) | 224(0.604) | 300(0.525) | 0.018 | NS | 1.37(1.05-1.79) |
|  |  | AA | 104(0.282) | 181(0.318) | 0.242 | NS | 0.84(0.63-1.12) | 107(0.288) | 181(0.318) | 0.352 | NS | 0.87(0.65-1.16) |
|  | rs1017119 | C | 57(0.076) | 72(0.064) | 0.283 | NS | 1.21(0.84-1.74) | 40(0.054) | 72(0.064) | 0.394 | NS | 0.84(0.56-1.25) |
|  |  | T | 689(0.924) | 1060(0.936) | 0.283 | NS | 0.82(0.57-1.17) | 700(0.946) | 1060(0.936) | 0.394 | NS | 1.18(0.79-1.77) |
|  |  | CC | 1(0.003) | 3(0.005) | 0.546 | NS | 0.50(0.05-4.86) | 2(0.005) | 3(0.005) | 0.983 | NS | 1.02(0.17-6.13) |
|  |  | CT | 55(0.147) | 66(0.117) | 0.167 | NS | 1.31(0.89-1.92) | 36(0.973) | 66(0.117) | 0.354 | NS | 0.81(0.53-1.25) |
|  |  | TT | 317(0.850) | 497(0.878) | 0.213 | NS | 0.78(0.53-1.14) | 332(0.022) | 497(0.878) | 0.367 | NS | 1.21(0.79-1.84) |
|  | rs7027797 | C | 20(0.027) | 29(0.025) | 0.818 | NS | 1.07(0.60-1.90) | 27(0.004) | 29(0.025) | 0.175 | NS | 1.44(0.84-2.45) |
|  |  | T | 716(0.973) | 1111(0.975) | 0.818 | NS | 0.93(0.52-1.66) | 717(0.964) | 1111(0.975) | 0.175 | NS | 0.69(0.40-1.18) |
|  |  | CT | 20(0.055) | 29(0.051) | 0.816 | NS | 1.07(0.59-1.92) | 27(0.073) | 29(0.051) | 0.168 | NS | 1.46(0.85-2.50) |
|  |  | TT | 348(0.945) | 541(0.949) | 0.816 | NS | 0.93(0.51-1.67) | 345(0.927) | 541(0.949) | 0.168 | NS | 0.68(0.39-1.17) |

Pc, P Bonferroni correction. OR, odds ratio. CI, confidence interval. NS, no significant different

**Supplementary Table 7 Main effects of C3 CNVs on clinical feature risk of BD**

| Clinical features | Copy  number | BD with | | BD without | | P value | Pc value | OR (95% CI) |
| --- | --- | --- | --- | --- | --- | --- | --- | --- |
|  |  | N | % | N | % |  |  |  |
| Genital ulcer |  | N=489 | | N=575 |  |  |  |  |
|  | <2 | 11 | 2.2 | 13 | 2.3 | 0.99 | NS | 0.99(0.44-2.24) |
|  | =2 | 443 | 90.6 | 524 | 91.1 | 0.76 | NS | 0.93(0.61-1.42) |
|  | >2 | 35 | 7.2 | 38 | 6.6 | 0.72 | NS | 1.08(0.67-1.75) |
| Skin lesions |  | N=379 | | N=685 |  |  |  |  |
|  | <2 | 9 | 2.4 | 15 | 2.2 | 0.84 | NS | 1.08(0.47-2.51) |
|  | =2 | 346 | 91.3 | 626 | 91.4 | 0.95 | NS | 0.98(0.63-1.54) |
|  | >2 | 24 | 6.3 | 44 | 6.4 | 0.95 | NS | 0.98(0.58-1.64). |
| Arthritis |  | N=164 | | N=900 |  |  |  |  |
|  | <2 | 5 | 3.0 | 21 | 2.3 | 0.58 | NS | 1.31(0.48-3.54) |
|  | =2 | 142 | 86.6 | 813 | 90.3 | 0.14 | NS | 0.69(0.41-1.13) |
|  | >2 | 17 | 10.4 | 66 | 7.4 | 0.18 | NS | 1.46(0.83-2.56) |
| Positive pathergy reaction |  | N=283 | | N=781 |  |  |  |  |
|  | <2 | 6 | 2.1 | 18 | 2.3 | 0.85 | NS | 0.91(0.36-2.33) |
|  | =2 | 256 | 90.5 | 708 | 90.6 | 0.92 | NS | 0.97(0.61-1.55) |
|  | >2 | 21 | 7.4 | 55 | 7.1 | 0.83 | NS | 1.05(0.62-1.78) |

BD, Behcet’s disease; OR, odds ratio; CI, confidence interval

**Supplementary Table 8 Main effects of C5 CNVs on clinical feature risk of BD**

| Clinical features | Copy  number | BD with | | BD without | | P value | Pc value | OR (95% CI) |
| --- | --- | --- | --- | --- | --- | --- | --- | --- |
|  |  | N | % | N | % |  |  |  |
| Genital ulcer |  | N=486 | | N=567 |  |  |  |  |
|  | <2 | 16 | 3.3 | 18 | 3.1 | 0.91 | NS | 1.04(0.52-2.06) |
|  | =2 | 445 | 91.5 | 520 | 91.7 | 0.93 | NS | 0.98(0.63-1.52) |
|  | >2 | 25 | 5.2 | 29 | 5.2 | 0.98 | NS | 1.00(0.58-1.74) |
| Skin lesions |  | N=377 | | N=676 |  |  |  |  |
|  | <2 | 11 | 2.9 | 24 | 3.5 | 0.58 | NS | 0.81(0.39-1.68) |
|  | =2 | 344 | 91.2 | 619 | 91.5 | 0.85 | NS | 0.96(0.61-1.50) |
|  | >2 | 22 | 5.9 | 33 | 5.0 | 0.50 | NS | 1.20(0.69-2.10) |
| Arthritis |  | N=164 | | N=889 |  |  |  |  |
|  | <2 | 7 | 4.2 | 21 | 2.3 | 0.16 | NS | 1.84(0.77-4.40) |
|  | =2 | 147 | 89.6 | 799 | 89.8 | 0.92 | NS | 0.97(0.56-1.68) |
|  | >2 | 10 | 6.2 | 69 | 7.9 | 0.45 | NS | 0.77(0.38-1.53) |
| Positive pathergy reaction |  | N=281 | | N=772 |  |  |  |  |
|  | <2 | 12 | 4.2 | 23 | 2.9 | 0.30 | NS | 1.45(0.71-2.96) |
|  | =2 | 255 | 90.7 | 714 | 92.5 | 0.35 | NS | 0.79(0.49-1.29) |
|  | >2 | 14 | 5.1 | 35 | 4.6 | 0.76 | NS | 1.10(0.58-2.08) |

BD, Behcet’s disease; OR, odds ratio; CI, confidence interval

Supplementary Table 9 Main effects of rs408290 /C3 on clinical feature risk of BD

| Clinical features | Genotype | BD with | | | BD without | | P value | Pc value | OR (95% CI) |
| --- | --- | --- | --- | --- | --- | --- | --- | --- | --- |
|  |  | N | | % | N | % |  |  |  |
| Genital ulcer |  | N=541 | | | N=407 |  |  |  |  |
|  | CC | 310 | 57.3 | | 231 | 56.7 | 0.86 | NS | 1.02(0.78-1.32) |
|  | CG | 162 | 29.9 | | 119 | 29.2 | 0.81 | NS | 1.03(0.78-1.37) |
|  | GG | 69 | 12.8 | | 57 | 14.1 | 0.57 | NS | 0.89(0.61-1.30) |
|  | G allele | 276 | 25.5 | | 211 | 25.9 | 0.83 | NS | 0.97(0.79-1.20) |
| Skin lesions |  | N=417 | | | N=531 |  |  |  |  |
|  | CC | 249 | 59.7 | | 297 | 55.9 | 0.24 | NS | 1.16(0.90-1.51) |
|  | CG | 125 | 29.9 | | 174 | 32.7 | 0.35 | NS | 0.87(0.66-1.15) |
|  | GG | 43 | 10.4 | | 60 | 11.4 | 0.62 | NS | 0.90(0.59-1.36) |
|  | G allele | 208 | 24.9 | | 276 | 25.9 | 0.60 | NS | 0.94(0.76-1.16) |
| Arthritis |  | N=135 | | | N=813 |  |  |  |  |
|  | CC | 78 | 57.7 | | 473 | 58.1 | 0.93 | NS | 0.98(0.68-1.42) |
|  | CG | 40 | 29.6 | | 243 | 29.8 | 0.95 | NS | 0.98(0.66-1.47) |
|  | GG | 17 | 12.7 | | 97 | 12.1 | 0.82 | NS | 1.06(0.61-1.84) |
|  | G allele | 72 | 26.6 | | 406 | 24.9 | 0.55 | NS | 1.09(0.81-1.46) |
| Positive pathergy reaction |  | N=261 | | | N=687 |  |  |  |  |
|  | CC | 150 | | 57.4 | 398 | 56.6 | 0.89 | NS | 0.98(0.73-1.30) |
|  | CG | 77 | | 29.5 | 206 | 29.9 | 0.88 | NS | 0.97(0.71-1.34) |
|  | GG | 34 | | 13.1 | 83 | 13.5 | 0.69 | NS | 1.09(0.71-1.67) |
|  | G allele | 140 | | 26.8 | 374 | 27.2 | 0.86 | NS | 0.98(0.78-1.23) |

BD, Behcet’s disease; OR, odds ratio; CI, confidence interval

Supplementary Table 10 Main effects of rs2269067/C5 on clinical feature risk of BD

| Clinical features | Genotype | BD with | | | BD without | | P value | Pc value | OR (95% CI) |
| --- | --- | --- | --- | --- | --- | --- | --- | --- | --- |
|  |  | N | | % | N | % |  |  |  |
| Genital ulcer |  | N=559 | | | N=421 |  |  |  |  |
|  | CC | 21 | 3.7 | | 17 | 4.0 | 0.82 | NS | 0.92(0.48-1.78) |
|  | CG | 156 | 27.9 | | 113 | 26.8 | 0.71 | NS | 1.05(0.79-1.40) |
|  | GG | 382 | 68.4 | | 291 | 69.2 | 0.79 | NS | 0.96(0.73-1.26) |
|  | G allele | 922 | 82.4 | | 689 | 81.8 | 0.71 | NS | 1.04(0.82-1.31) |
| Skin lesions |  | N=436 | | | N=544 |  |  |  |  |
|  | CC | 21 | 4.8 | | 25 | 4.5 | 0.87 | NS | 1.05(0.58-1.90) |
|  | CG | 109 | 25.0 | | 160 | 29.4 | 0.12 | NS | 0.80(0.60-1.06) |
|  | GG | 306 | 70.2 | | 359 | 66.1 | 0.16 | NS | 1.21(0.92-1.59) |
|  | G allele | 697 | 79.9 | | 897 | 82.4 | 0.15 | NS | 0.84(0.67-1.06) |
| Arthritis |  | N=140 | | | N=840 |  |  |  |  |
|  | CC | 7 | 5.0 | | 32 | 3.8 | 0.50 | NS | 1.32(0.57-3.07) |
|  | CG | 36 | 25.7 | | 205 | 24.4 | 0.73 | NS | 1.07(0.71-1.61) |
|  | GG | 97 | 69.3 | | 603 | 71.8 | 0.54 | NS | 0.88(0.60-1.30) |
|  | G allele | 238 | 85.0 | | 1375 | 81.8 | 0.20 | NS | 1.25(0.88-1.78) |
| Positive pathergy reaction |  | N=264 | | | N=716 |  |  |  |  |
|  | CC | 10 | | 3.7 | 28 | 3.9 | 0.93 | NS | 0.96(0.46-2.02) |
|  | CG | 66 | | 25.0 | 180 | 25.1 | 0.96 | NS | 0.99(0.71-1.37) |
|  | GG | 188 | | 71.3 | 508 | 71.0 | 0.93 | NS | 1.01(0.74-1.38) |
|  | G allele | 450 | | 85.2 | 1185 | 82.7 | 0.19 | NS | 1.20(0.91-1.58) |

BD, Behcet’s disease; OR, odds ratio; CI, confidence interval

Supplementary Table 11 Main effects of C3 CNVs on clinical feature of VKH syndrome

| Clinical features | Copy  number | VKH with | | VKH without | | P value | Pc value | OR (95% CI) |
| --- | --- | --- | --- | --- | --- | --- | --- | --- |
|  |  | N | % | N | % |  |  |  |
| Headache |  | N=224 | | N=835 |  |  |  |  |
|  | <2 | 7 | 3.1 | 24 | 2.8 | 0.84 | NS | 1.09(0.46-2.56) |
|  | =2 | 198 | 88.4 | 740 | 88.6 | 0.92 | NS | 0.97(0.61-1.55) |
|  | >2 | 19 | 8.5 | 71 | 8.6 | 0.99 | NS | 0.99(0.58-1.69) |
|  |  | N=132 | | N=927 |  |  |  |  |
| Tinnitus | <2 | 6 | 4.5 | 27 | 2.9 | 0.31 | NS | 1.58(0.64-3.92) |
|  | =2 | 113 | 85.6 | 832 | 89.7 | 0.15 | NS | 0.67(0.40-1.15) |
|  | >2 | 13 | 9.9 | 68 | 7.4 | 0.31 | NS | 1.38(0.74-2.57) |
| Vitiligo |  | N=28 | | N=1031 |  |  |  |  |
|  | <2 | 1 | 3.5 | 25 | 2.4 | 0.69 | NS | 1.49(0.19-11.4) |
|  | =2 | 25 | 89.2 | 935 | 90.6 | 0.80 | NS | 0.85(0.25-2.88) |
|  | >2 | 2 | 7.3 | 71 | 7.0 | 0.95 | NS | 1.04(0.24-4.47) |
| Alopecia and Poliosis |  | N=16 | | N=1043 |  |  |  |  |
|  | <2 | 1 | 6.2 | 29 | 2.8 | 0.40 | NS | 2.33(0.29-18.2) |
|  | =2 | 14 | 87.6 | 935 | 89.7 | 0.78 | NS | 0.80(0.18-3.60) |
|  | >2 | 1 | 6.2 | 79 | 7.5 | 0.84 | NS | 0.81(0.10-6.23) |

VKH, Vogt–Koyanagi–Harada syndrome; OR, odds ratio; CI, confidence interval

Supplementary Table 12 Genetic effect on BD and VKH syndrome of C3 and C5 polymorphisms

| Gene | SNP | Power for BD | Power for VKH |
| --- | --- | --- | --- |
| C3 | rs408290 | 0.99 | 0.65 |
|  | rs7951 | 0.62 | 0.89 |
|  | rs2250656 | 0.97 | 0.91 |
|  | rs432001 | 0.52 | 0.98 |
|  | rs344555 | 0.92 | 0.67 |
|  | rs2241394 | 0.11 | 0.07 |
| C5 | rs7040033 | 0.88 | 0.92 |
|  | rs1017119 | 0.52 | 0.26 |
|  | rs7027797 | 0.06 | 0.50 |
|  | rs2269067 | 0.99 | 0.18 |

BD, Behcet’s disease;VKH, Vogt–Koyanagi–Harada syndrome

Supplementary Table 13 Genetic effect on BD and VKH syndrome of C3, C5, C6, C7, C8A, C8B and C9 CNVs

| CNV | Power for BD | Power for VKH |
| --- | --- | --- |
| C3 | 0.99 | 0.99 |
| C5 | 0.99 | 0.99 |
| C6 | 0.81 | 0.35 |
| C7 | 0.78 | 0.15 |
| C8A | 0.99 | 0.99 |
| C8B | 0.94 | 0.86 |
| C9 | 0.89 | 0.82 |

BD, Behcet’s disease;VKH, Vogt–Koyanagi–Harada syndrome
